# Is RNA-dependent RNA polymerase essential for transposon control?

**DOI:** 10.1186/1752-0509-5-104

**Published:** 2011-06-29

**Authors:** Anton Crombach, Paulien Hogeweg

**Affiliations:** 1 Theoretical Biology and Bioinformatics Group, Utrecht University, Padualaan 8, 3584 CH Utrecht, The Netherlands; 2 EMBL/CRG Systems Biology Research Unit, Centre for Genomic Regulation (CRG), UPF, Dr Aiguader 88, 08003 Barcelona, Spain

## Abstract

**Background:**

Eukaryotes use RNA interference and RNA-based epigenetic regulation to control transposon activity. In the standard pathways of RNA-based transcriptional and post-transcriptional silencing the protein complex RNA-dependent RNA polymerase (RdRP) plays a crucial role. However, alternative pathways that bypass RdRP have recently been described. Hence two important questions are: is RdRP truly a necessary component for transposon control, and are the alternative RNA-based strategies also capable of controlling transposable elements?

**Results:**

We have studied the interplay between host RNAi pathways and transposons using mathematical models. We show that the canonical RdRP-based model controls transposons tightly, mainly via the feedback of cytoplasmic small RNA amplification. Next, we consider two variants lacking RdRP and instead employing antisense transcription of transposons. We show that transposon activity is also controlled by the alternative pathways, although cytoplasmic small RNA amplification is absent. Instead, control occurs in the nucleus, through a feedback in the epigenetic regulation.

**Conclusions:**

Concluding, our models show that the control of transposon activity can be achieved by alternative pathways that lack RdRP and act through different feedback mechanisms. Thus, although RdRP activity is ubiquitous in eukaryotes, it need not be a general requirement for transposon control.

## Background

Transposons are found in virtually all eukaryotes. They are DNA sequences that have the ability to create copies of themselves in the genome. This copying activity has been linked to a range of deleterious mutations, for instance chromosomal aberrations, and faulty expression of genes [1]. On the other hand, transposable elements (TEs) also appear to have been recruited in various nuclear processes such as alternative splicing [2], telomere maintenance [3], and even transposon control [4]. Clearly, TEs are a powerful mutagenic agent and hosts need to regulate their self-copying activity.

The large diversity of TEs, their different sequences and copy strategies have resulted in a variety of host responses [5]. In this study we focus on two main components that are employed in many eukaryotes: transcriptional and post-transcriptional gene silencing (TGS and PTGS) via RNA interference (RNAi), that is to say via template matching small RNA molecules. Note that we are not considering the related Piwi-based defense against transposons that is predominantly active in the germ line of multicellular organisms.

PTGS takes place in the cytoplasm. A double-stranded RNA molecule (dsRNA) is cleaved into small interfering RNAs (siRNAs), 21-25 nucleotides long, by a protein of the Dicer family. Next, single siRNA are loaded onto Argonaute proteins, which are part of a RNA Induced Silencing Complex (RISC). RISC identifies complementary RNA transcripts and subsequently degrades them by cleavage. In this manner TE mRNAs cannot be translated and thus TE activity is inhibited. In many eukaryotes the silencing response is enhanced and sustained by amplifying the number of siRNAs by means of creating secondary siRNAs [6]. Complementary base pair matching of siRNA with mRNA recruits RNA directed RNA polymerase (RdRP, in this case primed RdRP) that synthesizes a complementary strand resulting in a new dsRNA [7].

In the nucleus TEs are silenced by TGS. Both DNA methylation and histone modification patterns lead to the inhibition of expression of the underlying DNA sequence. Here we focus on (di)methylation of histone 3 at lysine 9 (H3K9me) as the signal resulting in heterochromatization. Such modifications are initiated and maintained by small RNAs [8]. Schematically, the following process takes place: DNA is transcribed into RNA, which is used by RdRP to form dsRNA. This dsRNA is subsequently sliced by a Dicer protein that physically and functionally interacts with RdRP [9]. Small RNA is then loaded on an Argonaute protein in the RNA induced transcription silencing complex (RITS) [10]. This complex recruits methyltransferase, CLR, that methylates nearby histones [11]. Additionally the complex appears to recruit other chromatin modifying proteins such as SWI, which bind to H3K9 methylated nucleosomes, compacting them and thus inhibiting transcription of the DNA [12]. Though the activity of RITS is mostly shown to operate in *cis *[12], we assume *trans*-effects as well [13].

In both above described silencing processes the protein complex RdRP appears to be a crucial component: it is required for the formation of dsRNA and for sustaining the cytoplasmic silencing response. In lower eukaryotes this role is fulfilled by the 'canonical' RdRP complex. In higher eukaryotes, such as fly (*D. melanogaster*) and mammals (mouse and human), canonical RdRP has not been found [14,15], yet various functional homologs have been experimentally characterized [16-19]. An important functional homolog is Elp1, as it is present in all eukaryotes and has been described to perform the task of canonical RdRP [17]. As Elp1 was discovered several years after canonical RdRP, various alternatives to the formation of dsRNA had been suggested and/or observed [20-22]. Thus even though now it is known that RdRP activity is present in all eukaryotes that are capable of RNAi [23,24], we should take into account the existence of alternative pathways. Hence an interesting question is how essential RdRP activity is to transposon silencing. Is it a crucial protein complex with respect to TE control, or are the other RNA processing pathways also capable of silencing transposable elements? In this study we aim to shed light on this matter by modeling RNAi-based silencing of transposons in the nucleus and cytoplasm, i.e. TGS and PTGS. We compare an RdRP-based mechanism, as described above, to two alternative mechanisms lacking RdRP.

If we assume RdRP is absent, an alternative way of generating dsRNA must be present and no siRNA amplification will occur. As mentioned before, several dsRNA formation strategies have been hypothesized and/or observed: convergent and divergent transcription from both strands [20], *trans*-acting natural antisense transcripts [22] and hairpins due to inverted repeats [20,21]. Considering the increasing evidence that antisense transcription is both widespread and associated with epigenetic silencing [13,25], we assume in both alternative scenarios dsRNA production via sense-antisense duplex formation in the nucleus. The two alternative pathways differ in the formation of cytoplasmic dsRNA. In the 'antisense' variant, we include antisense RNA (asRNA) transport to the cytoplasm and cytoplasmic sense-antisense dsRNA formation, while in the 'hairpin' variant we do not have such extra transport and dsRNA originates from hairpin formation of cytoplasmic mRNA.

Thus we approach the question whether RdRP is a necessary component in transposon control by comparing an RdRP-based model (the RdRP model) to two antisense-based models (the antisense and hairpin model). Specifically, we address this question in terms of active and silenced transposons, and abundance of TE products in the cytoplasm. Furthermore, we study the robustness of model behavior against parameter changes and how each of the models accomplishes transposon control by means of feedback loops.

## Results and Discussion

We studied mathematical models describing the dynamics of TEs, euchromatin and heterochromatin formation and cytoplasmic RNAi-based TE silencing. We report on three models composed of ordinary differential equations, which we introduce below (see also Methods and Additional File 1). In addition, we have checked that the results are independent of the modeling formalism by comparing ODE results against results from the stochastic versions of the models (see Additional File 2).

**Figure 1:**
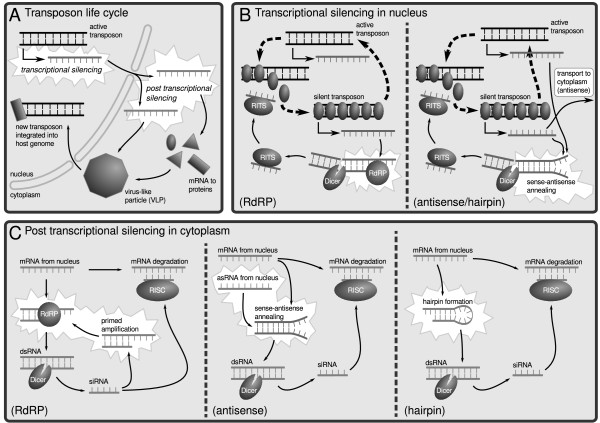
**Schematic depiction of TE control in the different models**. A. Transposon dynamics. Active TEs are transcribed and may integrate new TE copies into the host genome via several steps in the cytoplasm. B. Transcriptional silencing of TEs. Short transcripts are produced from heterochromatic regions of the genome, and these are processed into small RNAs (left panel). Alternatively, antisense RNA is produced from silenced TEs, which base pairs with mRNA and is then processed into small RNAs (right panel). In both cases small RNAs assist in heterochromatin formation. C. Post-transcriptional silencing of TEs. In the left panel, dsRNA is formed by RdRP from mRNA. In the middle panel, dsRNA is the result of sense-antisense annealing, and in the right panel mRNA hairpin formation leads to dsRNA. In all models the resulting dsRNA is cleaved into small RNAs. These small RNAs guide the degradation of base pair matching mRNA by RISC, and prime mRNA for dsRNA formation by RdRP (left panel only).

The RdRP model describes the standard RNAi-based TE silencing mechanism as elaborated in the Introduction (Figure 1). The other two models realize alternative pathways that do not require RdRP as described in the Introduction (Figure 1). In short, both alternative models enable dsRNA formation in the nucleus and cytoplasm, but in contrast to the RdRP model, they lack amplification of siRNAs in the cytoplasm. Furthermore, throughout the text we refer explicitly to retrotransposons with explicit stages in the cytoplasm (virus-like particles). However, we note that the underlying mathematics describe in general terms a TE with an intermediate stage in the cytoplasm. Thus the models apply to a wider range of TEs than only retrotransposons.

We compared three different TE control mechanisms by studying the scenario in which a single transposon invades a new genome: *T_act _*= 1.0 at time = 0. One can also view this as the re-activation of a TE already present in the host genome. Per model we performed 10000 simulations, and in each simulation each parameter value was picked randomly from a 100-fold range of a reference value (Table 1). In this manner we were able to assess the behavior of the models over a wide range of parameter combinations. We followed the number of active TEs (*T_act_*), chromatin-silenced TEs (*T_sil_*) and the number of virus-like particles in the cytoplasm (VLP) over a simulation time of 2 years and the final numbers of each of these observables we use in our comparison. In order to quantify how well a host controls its transposons, we take a single, rather strict condition that if VLP *<*1.0, we regard the host to be in control.

**Table 1:** Overview of parameters, their description and default value

**Par**.	Description	Value	Units
*j*	Integration of new transposon	0.1	hr^-1^
*f*	Fraction of successful integration	0.1	-
*v_ta_*	Transcription of active transposons	16	hr^-1^
*v_ts_*	Transcription of silenced transposons	1.6	hr^-1^
*t_m_*	Export of mRNA from nucleus	0.45	hr^-1^
*t_an_*	Export of asRNA from nucleus*^a^*	0.45	hr^-1^
*q*	VLP production (proteins etc)	1 · 10^-5^	#mol^-1 ^hr^-1^
*d_v_*	Decay of VLP	2.0	hr^-1^
*u*	Activation of silenced transposon	0.02	hr^-1^
*h_b_*	Basal heterochromatin formation	0.01	hr^-1^
*h_s_*	siRNA induced heterochromatin formation	0.001	#mol^-1 ^hr^-1^
*p_n_*	Rate of dsRNA synthesis from nuclear RNA	0.002	hr^-1^
*p_nx_*	dsRNA synthesis from mRNA and asRNA*^a, h^*	2 · 10^-4^	#mol^-1 ^hr^-1^
*d_r_*	Decay rate nuclear RNA	0.28	hr^-1^
*p_c_*	Rate of dsRNA synthesis from mRNA	0.002	hr^-1^
*p_cx_*	dsRNA synthesis from mRNA and asRNA*^a^*	2.5 · 10^-4^	#mol^-1 ^hr^-1^
*p_cxx_*	dsRNA synthesis from hairpin mRNA*^h^*	0.002	hr^-1^
*g*	Primed amplification rate	0.002	#mol^-1 ^hr^-1^
*b*	RISC activity	0.008	#mol^-1 ^hr^-1^
*d_m_*	Decay rate nuclear/cytoplasmic mRNA	0.14	hr^-1^
*g_s_*	Rate of dsRNA cleavage by Dicer	2.0	hr^-1^
*n*	Number of siRNAs cleaved from single dsRNA	10	-
*d_s_*	Decay siRNA	2.8	hr^-1^
*v_s_*	Degradation rate by RNAse	800	#mol^-1 ^hr^-1^
*k_s_*	Saturation constant	5.0	#mol

Units are number of molecules (#mol), and per hour (hr^-1^). Parameters marked in their description with *a *and/or *h *are used only in the antisense or hairpin models respectively. Parameter value sampling is performed by assuming a uniform distribution in the interval [0.1 _* _*parameter*, 10.0 _* _*parameter*] and drawing random values from this distribution. See Additional File 3 for references and estimations of parameter values.

### Assessing transposon activity

**Figure 2:**
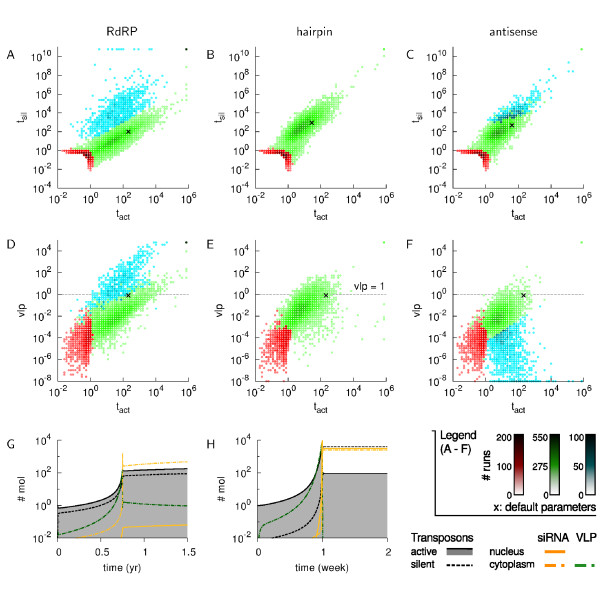
**Transposon activity of 10000 simulations at ***t *= 2 **years**. A, B, C. Two-dimensional histograms of active (*t_act_*) against silenced (*t_sil_*) transposons. D, E, F. Two-dimensional histograms of active transposons against cytoplasmic virus-like particles (*vlp*). The cross (×) indicates the result of a run with default parameters (Table 1). The horizontal line indicates the condition *V LP <*1.0. A, D. RdRP model, the simulations are categorized in three clusters. Red indicates the "no invasion" cluster, green the "controlled" cluster and blue the "out of control" simulations. B, E. Hairpin model, two cluster are distinguished: the red "no invasion" and green "controlled" cluster. C, F. Antisense model, three clusters of simulations are present: red is "no invasion", green "controlled", and blue "low VLP". Note the log scale of both axes in all figures. G. Time plot of a single simulation using the default parameter values of the RdRP model (green cluster). H. Time plot of a single simulation from the antisense model (blue cluster).

The first observation we make, is that the RdRP model and the two antisense variants produce results in similar orders of magnitude, both with respect to active against silenced TEs, and active TEs against VLPs (Figure 2A-F). If we focus on Figure 2A-C, there is a typical grouping of points into two or three clusters. We observe a relatively small cluster (14% of the simulations in each model) with a total number of TEs smaller than 2 (*T_act _*+ *T_sil _≤ *2.0). Clearly, in these simulations the transposable element has hardly been able to copy itself within the two year time span. There are two underlying causes. the TE is not capable of increasing its copy number due to a very strict silencing regime of the host or the TE has an intrinsic low copy rate, i.e. it is a rare event that simply occurred only once. We refer to this group of simulations as cluster 1, or the "no invasion" cluster.

In all models, the main cluster is an elliptically-shaped group of runs (2nd, or green cluster in Figure 2). In these runs, the number of active and/or silent elements ranges from *>*2 to ~10^4^. Thus TEs invaded the host genome, but if we look at the level of VLPs, we also find most runs satisfy the condition of VLP *<*1.0. We find that in the RdRP model the host silences a fraction of 0.90, while in the antisense model a fraction of 0.98 is under control and in the hairpin model a fraction of 0.91 (Figure 2D-F and Table 2). Therefore the large majority of parameter combinations, even if they are generated at random, results in host control of the TE invasion.

**Table 2:** Descriptive statistics of the three models

model	RdRP			Hairpin		antisense		
cluster	red	green	blue	red	green	red	green	blue
median *T_act_*	0.70	36.75	58.52	0.65	7.64	0.65	7.30	25.28
median *T_sil_*	0.51	19.43	1.63e4	0.54	372.57	0.55	110.21	2866.41
median VLP	1.1e-4	0.01	4.62	1.0e-4	0.05	0.9e-4	0.01	19.2e-4
frac. runs	0.14	0.64	0.10	0.14	0.78	0.14	0.73	0.13
frac. *VLP <*1.0	1.00	0.90	0.33	1.00	0.91	1.00	0.98	1.00

Per cluster (see Figure 2) the median active TEs (*T_act_*), silent TEs (*T_sil_*) and virus-like particles (VLP) are given. In addition, per cluster its size is given as a fraction of total runs (runs/10000), and its fraction of runs that satisfy VLP*<*1.0.

Furthermore, here the RdRP model differs in two respects from both alternative variants. Firstly, in the RdRP model the range of active TEs is a magnitude larger than in the antisense and hairpin model and there is a markedly smaller number of silenced TEs per active TEs. If we express this in numbers, the RdRP model has active TEs in the range [1, 10^4^], while the two alternatives show a range of [1, 10^3^]. Further, silenced TEs lie in the RdRP model within [0.1, 10^3^], and in the alternative models within [10, 10^5^] (see also Table 2).

Secondly, the RdRP and antisense model have an additional third cluster in which a high number of silenced TEs has accumulated relatively to the number of active TEs. However, there is a distinct difference between the two models with respect to the levels of VLP in the cytoplasm. In the RdRP model, this third cluster corresponds to high levels (*>*1.0) of VLPs (see the 3rd, blue cluster in Figure 2D). That is to say, given that VLPs are the precursors of new transposon copies in the host' genome, the high VLP levels imply TEs continue to be readily integrated into the genome. The majority of runs in this cluster have in common that the primed amplification rate is low (*g *≪ 0.002). In other words, due to an insufficiently strong feedback in siRNA production, the RdRP-based silencing response is inadequate for the control of transposable elements.

In contrast to the high levels of VLPs in the RdRP model, the antisense model shows the opposite behavior (Figure 2F). Along the entire range of active TEs there is a subset of runs with extremely low VLP levels. In these runs TEs replicate fast, which results in an overshoot of active TEs as the host silencing threshold is passed. Subsequently, a large number of TEs are silenced (*T_sil _*≈ 10^4^) and thus a relatively large number of asRNA is available for silencing TEs.

This behavior does not occur in the hairpin variant. The main difference between the antisense and hairpin model is that in the antisense model the asRNA is also available in the cytoplasm, thus resulting in the observed low VLP levels.

Given the above observations, we come to the interesting conclusion that in all three models the large majority of parameter settings result in invading TEs being silenced (controlled) by the host. At least within our modeling framework the two pathways based on antisense transcription appear to be viable, robust alternatives to the standard RdRP-based pathway. In other words, our results suggest RdRP does not need to be as essential for silencing transposons as has been implicitly assumed up to now.

### The mechanics of TE control

In order to appreciate how the two antisense-based variants allow for equally robust TE control as the RdRP model, we need to understand the underlying mechanism of this silencing. How are TEs exactly silenced in the three models?

First of all, all three models share an amplification loop of TE growth. If left unchecked, exponential growth is realized via transcription of transposons, several stages in the cytoplasm and the subsequent integration of a new transposon in the host genome.

Secondly, what sets the RdRP model apart from the antisense variants is that there is an amplification of siRNAs in the cytoplasm. The production of siRNA promotes the recruitment of mRNA into dsRNA formation, hence leading to more small RNAs (Figure 1b). In this manner the expression of TEs is controlled post-transcriptionally in the cytoplasm. The process is analogous to transgene silencing [26], and as this feedback loop depends on the presence of RdRP, it is not found in the two alternative models.

**Figure 3:**
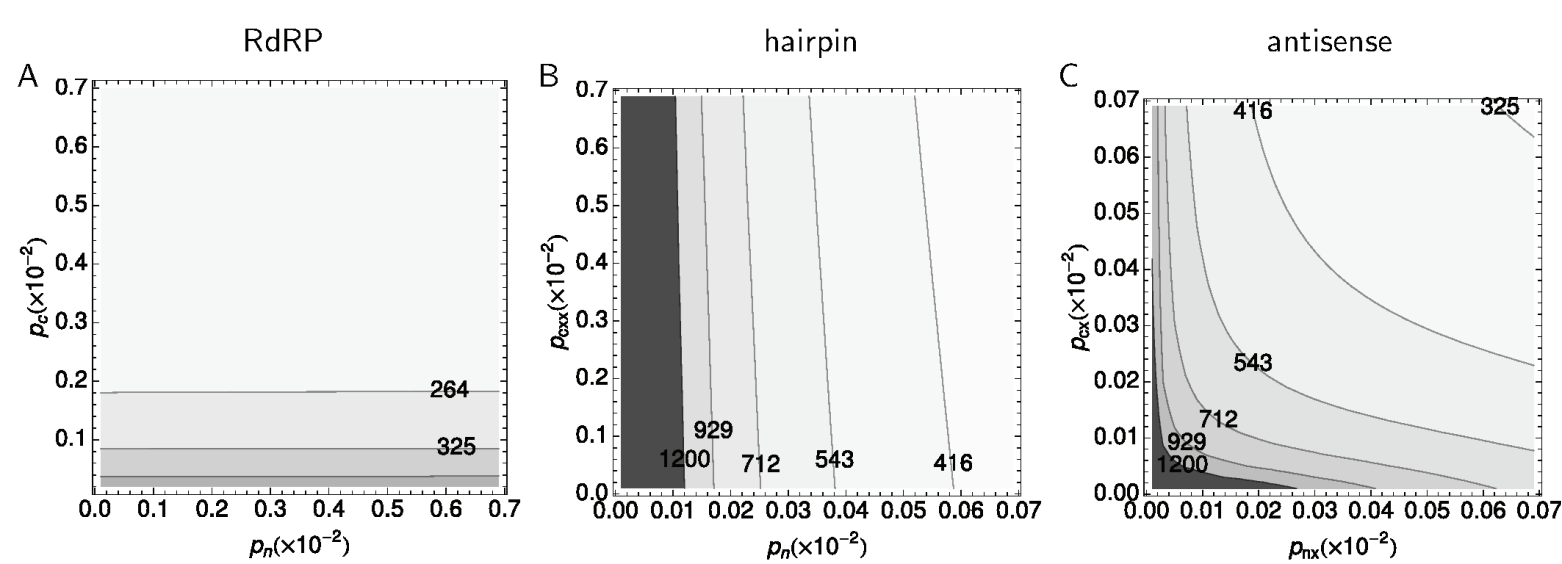
**Recruitment for silencing**. The panels display a contour map of the total number of TEs at *t *= 2 yr. Nuclear dsRNA formation is on the × axis, while cytoplasmic dsRNA formation is on the y axis. Note the different scale of the axis between the figures. A. RdRP model. There is a distinct gradient of total number of TEs along the y axis. B. Hairpin model, with a clear gradient of total number of TEs along the × axis. C. Antisense model, with a gradient of total number of TE along both axes.

In contrast to the cytoplasmic control of the RdRP model, the two alternatives utilize a feedback loop in the nucleus via heterochromatization. As mentioned in the Introduction, heterochromatin is not equivalent to complete transcriptional silencing. Instead from genomic regions where TEs reside, RNA transcripts originate that are processed into small RNAs, which in turn guide the formation and expansion of heterochromatin into neighboring regions and other genomic locations with matching base pairs between the small RNA and the genomic DNA. That is to say, the small RNAs spread a silencing signal over the host genome. As more TEs become silenced in heterochromatin, the signal becomes stronger until an equilibrium is reached where active and silent transposons keep each other in check. In combination with sense-antisense duplex formation or hairpin formation in the cytoplasm, this nuclear-based control is sufficient for a robust silencing of TEs (Figure 1 and 3). To our knowledge this third, nucleus-based, feedback mechanism has not been acknowledged as a possibly important component in the control of transposable elements.

Finally, we test the influence of both silencing pathways on the total number of TEs by varying the recruitment parameters in the nucleus, *p*_*n*(*x*)_, and cytoplasm, *p*_*c*(*x*) _and *p_cxx_*. In agreement with the above observations, we find a clear distinction between the RdRP model and both antisense-based variants. In the RdRP model, cytoplasmic silencing dominates over nuclear silencing (Figure 3A); there is hardly any influence visible from heterochromatic silencing. In contrast, in our alternative models the nuclear-based pathway is crucial, as can be observed from the gradient along the *p_nx _*axis (Figure 3B, C). Moreover, we are able to distinguish between the antisense and hairpin variant. Due to the antisense transport from nucleus to cytoplasm in the antisense model, cytoplasmic silencing benefits as the number of silenced TEs increases. This allows tighter control of the TEs in the antisense model.

In summary, in the RdRP model cytoplasmic PTGS dominates, in contrast to the emphasis of TE control on the nuclear TGS mechanism in both antisense variants.

## Conclusions

We have presented an initial approach of modeling RNAi-based regulation of TEs, incorporating transposon dynamics, epigenetic silencing and cytoplasmic silencing. We have reported on an RdRP-based model and two asRNA-based variants. The main result is that these models have allowed us to question whether RdRP activity is an essential component of the TE silencing machinery and to assess the effectiveness of alternative pathways of TE control.

RdRP activity - either acting through canonical RdRP or Elp1-related RdRP - is ubiquitous among eukaryotes that employ RNAi. With respect to transposon control RdRP activity is considered essential for two reasons. Firstly, dsRNA formation is a crucial step in the functioning of both nuclear and cytoplasmic RNA-based silencing. Secondly, cytoplasmic siRNA amplification by RdRP is needed for sustained silencing. With regards to dsRNA formation, alternatives have been found through experimental approaches - antisense transcription [22] and hairpin formation [20,21] - which we used to formulate alternative mechanisms of small RNA based TE control. With regards to the amplification of small RNAs, a known alternative is the so-called ping-pong model of piwiRNA, which we did not address in this study. We propose a different, more indirect, amplification route that may contribute to TE control. Instead of amplification of siRNA in the cytoplasm, this amplification loop is based on (antisense) transcription of silenced TEs. The more TEs are silenced, the more that they are transcribed and the tighter host control becomes.

A striking difference we observed between cytoplasmic and nuclear silencing is the ratio between active and silent TEs. Cytoplasmic silencing lead to an approximate equal amount of active and silent TE copies, while nuclear control reached a ratio ~1 : 50 of active against silent TEs. These are such distinct results that it is tantalizing to hypothesize that one can distinguish between the different modes of TE control in plants and animals. However, we note that per TE family the host appears to apply different control measures, and small RNA based silencing is not the only means of control a host may employ [5].

We hope that further experimental research will be done to provide additional data to verify whether a 'nuclear', indirect feedback mechanism is actually employed in TE control. We would be interested in the mechanics of antisense RNA production with respect to Pol II and Elp1 [16,24], and the processing of sense-antisense pairs in both the nucleus and cytoplasm. These are important factors in silencing without RdRP, but to our knowledge have not been extensively characterized experimentally. Also, the role of transport of antisense transcripts and siRNAs, both between the cytoplasm and nucleus and within the nucleus has many unknowns. As mentioned, one important assumption in our model is *trans*-acting siRNAs in the nucleus. This process is only starting to be unraveled [13].

Finally, though we studied the RNAi pathways separately, it is likely that both RdRP and antisense-based dsRNA formation are active in a cell at any given moment. In this light, experimental research may be accompanied by modeling efforts to delineate the relative contribution of each pathway. We expect the outcome of such models to depend on the characteristics of the TE family under consideration and the host species the transposons are active in.

Concluding, this work provides an exploratory modeling approach to transposon dynamics and the subsequent silencing via RNAi in nucleus and cytoplasm. We have shown that, even though RdRP is present in all eukaryotes, there are viable alternative silencing pathways that are based on antisense transcription and asRNA transport combined with the feedback caused by heterochromatin formation.

## Methods

Each model consists of three components: transposon copying with intermediate stages in the cytoplasm, transcriptional silencing (TGS) via small RNA guided heterochromatin formation and post transcriptional silencing (PTGS) via small RNA mediated mRNA degradation. These processes have been subdivided into a set of pseudo-reactions, which are given in Table 3. Note that decay of TEs is not included, and as a consequence we study a system that is in a transient state, and not in an equilibrium.

**Table 3:** Reactions and their availability in the three models.

Transposon life cycle				
**Description**	**Reaction**	**RdRP**	**Antisense**	**hairpin**

VLP integration in genome		+	+	+
production of mRNA		+	+	+
production of other/antisense RNA		+	+	+
transport of mRNA		+	+	+
decay of mRNA		+	+	+
production of VLP		+	+	+
decay of VLP		+	+	+
VLP integration failed		*implicitly present*		
				
**Transcriptional gene silencing**				

**Description**	**Reaction**	**RdRP**	**Antisense**	**hairpin**

TE spontaneously silenced		+	+	+
TE silenced by RITS		+	+	+
TE activation		+	+	+
production of dsRNA by RdRP		+	-	-
dsRNA by sense-antisense base pairing		-	+	+
transport of antisense RNA		-	+	-
decay of other/antisense RNA		+	+	+
production of siRNA		+	+	+
decay of siRNA		+	+	+
				
**Post-transcriptional gene silencing**				

**Description**	**Reaction**	**RdRP**	**Antisense**	**hairpin**

production of dsRNA by RdRP		+	-	-
production of dsRNA from primed mRNA		+	-	-
dsRNA by sense-antisense base pairing		-	+	-
dsRNA by hairpin formation		-	-	+
degradation by RISC		+	+	+
decay of mRNA		+	+	+
decay of antisense RNA		-	+	-
production of siRNA		+	+	+
decay of siRNA		+	+	+

In the first column a short description of the reaction is given, in the second column the pseudo-reactions are given as a formula, followed by three columns indicating the presence (+) and absence (*-*) of each reaction in the different models. Note that we do not consider the decay of TEs, and as a consequence we study a system that is in a transient state, not an equilibrium. Symbol legend: *T_act _*active TE, *T_sil _*silent TE, *V *virus-like particle, *M_n _*nuclear mRNA, *M_c _*cytoplasmic mRNA, *R_n _*nuclear other/antisense RNA, *R_c _*cytoplasmic antisense RNA, *D_n _*nuclear dsRNA, *D_c _*cytoplasmic dsRNA, *S_n _*nuclear siRNA, *S_c _*cytoplasmic siRNA.

Below we highlight a few characteristics of our modeling approach. Firstly, transposon copying has been modeled according to the life cycle of retrotransposons, with a stage in the cytoplasm including a virus-like particle (VLP). This may seem rather specific, however the mathematical equations describing the TE life cycle allow for a more general interpretation: the equations capture a general reproductive cycle with intermediate steps (protein products etc) in the cytoplasm. Thus the model can be understood as describing a generalized TE with an intermediate phase in the cytoplasm.

Secondly, we only take into account fully functional transposable elements, and we do not model defective or non-autonomous TEs. This modeling decision does not affect the generality of the models as the omission of these passive transposable elements provides us with a worst case scenario: every TE is capable of reproduction and thus every new copy of a transposon "puts host control under more pressure".

Finally, from the reactions as given in Table 3 we derived for each model the set of ordinary differential equations (ODEs) and the corresponding stochastic (Gillespie) reactions. These are given in Additional File 1 and 2. Furthermore, the default parameter values are listed in Table 1 and their derivation in Additional File 3.

### Programs used

The ODE equations were numerically integrated using MATLAB, and subsequently analyzed using R (http://www.r-project.org), Python (http://www.python.org) and Gnuplot (http://www.gnuplot.info).

Stochastic versions of the models were simulated using in-house developed software, which is available upon request.

## List of abbreviations

Abbreviation Description

asRNA: antisense RNA; dsRNA: double-stranded RNA; mRNA: messenger RNA; PTGS: post-transcriptional gene silencing; RdRP: RNA-dependent RNA polymerase; RISC: RNA induced silencing complex; RITS: RNA induced transcription silencing; RNAi: RNA interference; TE: transposable element; TGS: transcriptional gene silencing; VLP: virus-like particle

## Authors' contributions

AC conceived of the study, analyzed the data and drafted the manuscript. PH participated in the design of the study, analyzed the data and helped to draft the manuscript. All authors read and approved the final manuscript.

## Supplementary Material

Additional file 1 **Model Description**. Model description in terms of mathematical equations. Click here for file

Additional file 2 **Stochastic Simulations**. A short description of the stochastic simulations of each of the models. Click here for file

Additional file 3 **Parameter Choice**. Additional information to elaborate on the choice of parameter value ranges. Click here for file
