# Supplementary material for: Is RNA-dependent RNA polymerase essential for transposon control?
Source: BMC Syst Biol. 2011 Jun 29;5:104. doi: 10.1186/1752-0509-5-104 (PMC3155503; doi:10.1186/1752-0509-5-104)
Supplement: Additional file 1 — Model Description. Model description in terms of mathematical equations. [file 1752-0509-5-104-S1.PDF]

Supporting Information 1: Model Description

RdRP model

Below the ODE representation of the RdRP-based model is described.

Transposons

$$T'_{act} = jfV + uT_{sil} - (h_b + h_s S_n)T_{act} \tag{1}$$

A fraction f of the virus-like particles manages to integrate at rate j a new transposon into the DNA of the host. Some transposons are activated u from the silenced state, while active transposons are silenced with a basal rate h_b and by small RNA h_s. We do not consider the decay of TE.

Silenced transposons

$$T'_{sil} = (h_b + h_s S_n)T_{act} - uT_{sil} \tag{2}$$

Silenced transposons originate from active ones, h_b and h_s, and may be activated at rate u again. Note that silent TE do not decay.

Nuclear mRNA

$$M'_n = v_{ta}T_{act} - t_m M_n - d_m M_n \tag{3}$$

Transcription of active transposons occurs with a rate v_{ta}. The resulting mRNA is either transported to the cytoplasm, t_m, or decays in the nucleus, d_m.

Nuclear other RNA

$$R'_n = v_{ts}T_{sil} - p_n R_n - d_r R_n \tag{4}$$

Silenced transposons produce RNA (untranslated transcripts) that is immediately processed by RdRP to dsRNA with rate p_n, or degraded d_r.

Nuclear dsRNA

$$D'_n = p_n R_n - g_s D_n \tag{5}$$

Nuclear dsRNA is produced from the transcripts of silenced transposons, p_n, and cleaved by Dicer, g_s. Note that in contrast to cytoplasmic RNA-based silencing we do not consider primed amplification.

Nuclear siRNA

$$S'_n = g_s n D_n - \frac{v_s S_n}{k_s + S_n} - d_s S_n - h_s S_n T_{act} \tag{6}$$

Nuclear small RNA is produced from dsRNA, g_s. The small RNAs are degraded enzymatically, v_s, and according to mass action d_s (see also [Groenenboom et al., 2005]). Next, nuclear siRNA are used by RITS in the heterochromatization of transposons, h_s.

Cytoplasmic mRNA

$$M'_c = t_m M_n - qM_c^2 - d_m M_c - p_c M_c - gS_c M_c - bS_c M_c \tag{7}$$

From the nucleus mRNA enters the cytoplasm with rate t_m. Here it is used in the production of VLP, q, or simply decays, d_m. With respect to cytoplasmic silencing mRNA is made into a dsRNA by RdRP, p_c. Also, RdRP-based amplification by priming with siRNA occurs, g. Furthermore, mRNA is subjected to RISC degradation, b.

Cytoplasmic dsRNA

$$D_c' = p_c M_c - g_s D_c + g S_c M_c \tag{8}$$

Cytoplasmic dsRNA is created from cytoplasmic mRNA, p_c and primed RNA g, and thereafter cleaved by Dicer, g_s.

Cytoplasmic siRNA

$$S_c' = g_s n D_c - \frac{v_s S_c}{k_s + S_c} - d_s S_c - b S_c M_c - g S_c M_c \tag{9}$$

Small RNAs are produced by cleavage of dsRNA by Dicer, and subsequently degraded by an RNAse, v_s, k_s and 'overflow' decay d_s. With respect to Groenenboom et al. [2005], this is one of their extensions to the basic pathway of RNAi. Small RNAs are integrated into RISC and facilitate degradation of mRNA, b. Note that we assume that small RNA are degraded as well in the process, which may be a more stringent constraint than biologically needed. Small RNAs also prime mRNA for synthesis of a second complementary strand by RdRP, g.

Virus-like particle

$$V' = q M_c^2 - jV - d_v V \tag{10}$$

Though we model transposon activity as if it is a retrotransposon that codes for a virus-like particle (VLP), it holds for all TEs that various proteins and intermediate steps are required to create a new DNA copy that can be integrated into the host genome. Thus the terms may be viewed as the general process of protein production etc. that is required for transposon activity. Throughout this report we refer to V as VLP, or VLP levels. We model the production of a new transposon, q, as a process with some cooperativity among mRNAs. This is most obvious in retrotransposons. In yeast, Ty families require two mRNA to form a dimer in order to produce a single DNA copy. The resulting VLP (or its load, a DNA) moves to the nucleus for integration, j. In addition we have decay of VLPs, d_v.

Alternative models (antisense and hairpin)

Below we list the equations that changed due to the absence of RdRP, with extra or changed terms in bold. We now assume silenced transposons produce antisense transcripts at a low level instead of unspecified RNA transcripts, and hence dsRNA may be formed from duplex formation of antisense RNA (asRNA) with mRNA. In the antisense model we allow this to occur both in the nucleus and cytoplasm, and in this model we also include transport of asRNA across the nuclear envelope. In the hairpin model there is no such asRNA transport present, and we assume cytoplasmic dsRNA is produced from hairpin formation of mRNA (see Eq. 15 and Eq. 18).

Nuclear mRNA

$$M_n' = v_{ta} T_{act} - t_m M_n - d_m M_n - \mathbf{p_{nx} R_n M_n} \tag{11}$$

Nuclear mRNA is recruited in the formation of dsRNA, p_{nx}.

Nuclear asRNA

$$R_n' = v_{ts} T_{sil} - \mathbf{p_{nx} R_n M_n} - d_r R_n - \mathbf{t_{an} R_n} \tag{12}$$

Silenced transposons produce asRNA that may combine with mRNA to dsRNA with rate p_{nx}. Also, asRNA is transported to cytoplasm, t_{an}.

Nuclear dsRNA

$$D_n' = \mathbf{p_{nx} R_n M_n} - g_s D_n \tag{13}$$

Nuclear dsRNA is produced from asRNA and mRNA at rate p_{nx}, and cleaved by Dicer, g_s.

Cytoplasmic mRNA (antisense model)

$$M_c' = t_m M_n - qM_c^2 - d_m M_c - \mathbf{p_{cx}R_cM_c} - bS_cM_c \tag{14}$$

The generation of dsRNA is based on mRNA and asRNA, p_{cx}. Also, there is no amplification of siRNAs via primed RdRP.

Cytoplasmic mRNA (hairpin model)

$$M_c' = t_m M_n - qM_c^2 - d_m M_c - \mathbf{p_{cxx}M_c} - bS_cM_c \tag{15}$$

The generation of dsRNA is based on hairpin mRNA, p_{cxx}. There is no amplification of siRNAs via primed RdRP.

Cytoplasmic asRNA (only in antisense model)

$$\mathbf{R_c'} = \mathbf{t_{an}R_n} - \mathbf{p_{cx}R_cM_c} - \mathbf{d_rR_c} \tag{16}$$

Cytoplasmic antisense RNA is imported from the nucleus, t_{an}, can be recruited for dsRNA, p_{cx}, and may decay d_r.

Cytoplasmic dsRNA (antisense model)

$$D_c' = \mathbf{p_{cx}R_cM_c} - g_sD_c \tag{17}$$

Cytoplasmic dsRNA is created from cytoplasmic asRNA and mRNA, p_{cx}, and thereafter cleaved by Dicer, g_s.

Cytoplasmic dsRNA (hairpin model)

$$D_c' = \mathbf{p_{cxx}M_c} - g_sD_c \tag{18}$$

Cytoplasmic dsRNA is created from cytoplasmic mRNA hairpins, p_{cxx}, and thereafter cleaved by Dicer, g_s.

Cytoplasmic siRNA

$$S_c' = g_s n D_c - \frac{v_s S_c}{k_s + S_c} - d_s S_c - bS_cM_c \tag{19}$$

As RdRP is absent, small RNAs in the cytoplasm are not subjected to primed amplification.

References

MAC Groenenboom, AFM Marée, and P Hogeweg. The RNA silencing pathway: the bits and pieces that matter. *PLoS Comput Biol*, 1(2):155–165, Jul 2005.
