# Supplementary material for: Is RNA-dependent RNA polymerase essential for transposon control?
Source: BMC Syst Biol. 2011 Jun 29;5:104. doi: 10.1186/1752-0509-5-104 (PMC3155503; doi:10.1186/1752-0509-5-104)
Supplement: Additional file 2 — Stochastic Simulations. A short description of the stochastic simulations of each of the models. [file 1752-0509-5-104-S2.PDF]

Supporting Information 2: Stochastic simulations

Stochastic formulation of the three models

In this study we have predominantly used a set of ordinary differential equations (ODE) to describe the behavior of transposons and host control. However, it is well known that for small numbers of molecules intrinsic noise may play an important role in the dynamics. In order to verify our results with respect to small numbers of transposons, VLP levels and other molecules we also design and study a stochastic version of our model using the stochastic simulation algorithm, or Gillespie algorithm [Gillespie, 1977]. Below we describe the pseudo-reactions of this approach. The reactions, with the corresponding parameters above the reaction arrows, are a straightforward translation from the original models as described in the Methods. Thus parameters are the same as used in the ODE model.

Transposon life cycle

$$T_{act} \xrightarrow{v_{ta}} T_{act} + M_n \qquad M_n \xrightarrow{d_m} \emptyset$$

$$T_{sil} \xrightarrow{v_{ts}} T_{sil} + R_n \qquad V \xrightarrow{j \cdot f} T_{act}$$

$$M_n \xrightarrow{t_m} M_c \qquad V \xrightarrow{j \cdot (1-f)} \emptyset$$

$$2M_c \xrightarrow{q} V \qquad V \xrightarrow{d_v} \emptyset$$

In the transposon life cycle we introduce one extra decay reaction. The decay of VLPs that fail to successfully integrate into the host genome is implicitly present in the original models, and needs to be explicitly modeled here.

Transcriptional gene silencing

$$T_{act} \xrightarrow{h_v} T_{sil} \qquad R_n + M_n \xrightarrow{p^*_{nx}} D_n$$

$$T_{act} + S_n \xrightarrow{h_s} T_{sil} \qquad R_n \xrightarrow{d_r} \emptyset$$

$$T_{sil} \xrightarrow{u} T_{act} \qquad D_n \xrightarrow{g_s} n\ S_n$$

$$R_n \xrightarrow{p_n} D_n \qquad S_n \xrightarrow{d_s,\ (v_s, k_s)} \emptyset$$

Post-transcriptional gene silencing

$$M_c \xrightarrow{p_c,\ p^*_{cxx}} D_c \qquad M_c + S_c \xrightarrow{b} \emptyset$$

$$M_c + R_c \xrightarrow{p^*_{cx}} D_c \qquad M_c \xrightarrow{d_m} \emptyset$$

$$M_c + S_c \xrightarrow{g} D_c \qquad R_c \xrightarrow{d_r} \emptyset$$

$$D_c \xrightarrow{g_s} n\ S_c \qquad S_c \xrightarrow{d_s,\ (v_s, k_s)} \emptyset$$

Both in TGS and PTGS the reactions with a star (*) are the alternative pathways of dsRNA formation, one via duplex formation of mRNA and asRNA and the other as hairpin formation of mRNA. Note that the decay of siRNA both in the nucleus and cytoplasm is composed of two reactions, one according to mass action kinetics (d_s), and one following saturating kinetics (v_s, k_s).

Transport of asRNA

$$R_n \xrightarrow{t^*_{an}} R_c$$

Transport of 'other' RNA, R_n, across the nuclear envelop is marked with a star (*) as we only consider it in the antisense model.

Results and discussion of stochastic models

We ran a small batch of simulations for each model. In figure S1 for each model 25 runs are shown. The final numbers of active and silent transposons coincide with the ODE results (Figure 2): the stochastic simulations end within the ODE cluster boundaries. Furthermore, we note that in stochastic simulations the variables are discrete – half a VLP does not exist – and therefore many simulations have zero or one VLP (Figure S1D,E,F). This is also clearly visible in the timeplots (Figure S1G,H,I). Finally, as was the case in the ODE simulations, in the stochastic simulations we observe that the RdRP model generates a rather equal amount of active and silent TEs, while in the two variants we observe a large pool of silent TEs in comparison to the active transposons.

References

DT Gillespie. Exact stochastic simulation of coupled chemical reactions. *Journal of Physical Chemisty*, 81:2340–61, 1977.

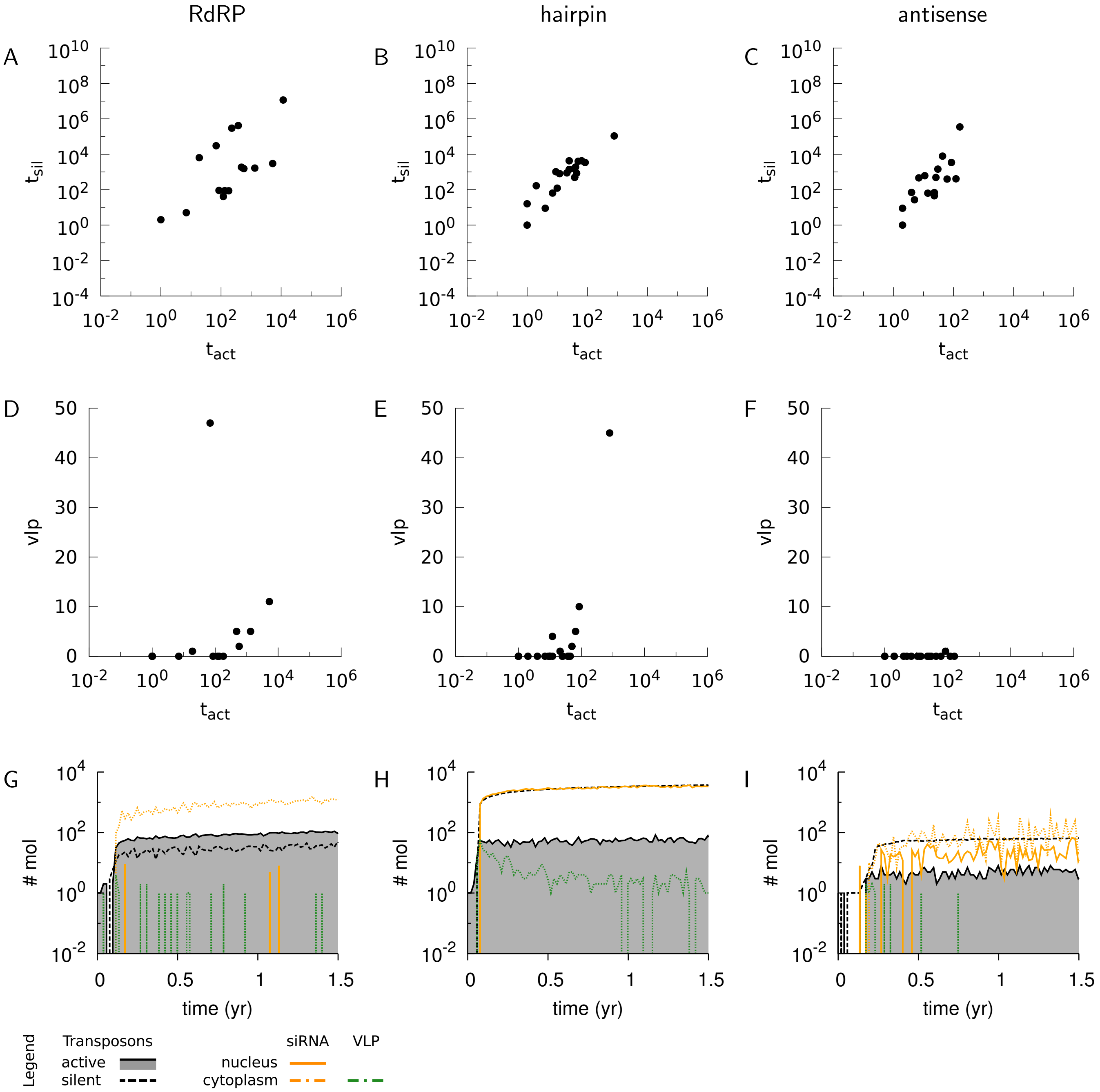

Figure S1 – Transposon activity of 25 simulations at t = 2 years. A,B,C. Scatter plot of active (t_{act}) against silenced (t_{sil}) transposons. Note the log scale of both axes. D,E,F. Scatter plot of active transposons against virus-like particles (vlp). G,H,I. Time plots, with a sampling rate of 1/week, of a randomly selected run for each model; RdRP, hairpin and antisense respectively.
