# Supplementary material for: Is RNA-dependent RNA polymerase essential for transposon control?
Source: BMC Syst Biol. 2011 Jun 29;5:104. doi: 10.1186/1752-0509-5-104 (PMC3155503; doi:10.1186/1752-0509-5-104)
Supplement: Additional file 3 — Parameter Choice. Additional information to elaborate on the choice of parameter value ranges. [file 1752-0509-5-104-S3.PDF]

Supporting information 3: Parameter Choice

Transposon life cycle The transcription rate of active transposons is estimated to be rather low: we take it a tenth of the mRNA influx of Groenenboom et al. [2005], that is $v_{ta} = 16$ hr^{-1}. Silent transposons are assumed to be transcribed only occasionally, thus at an even lower rate than active ones: we take their transcription rate a 10-fold lower ($v_{ts} = 1.6$ hr^{-1}).

Simple mass action mRNA transport is taken such that a ratio 1:3 between nucleus and cytoplasm is obtained in agreement with experimental observations [Jarmolowski et al., 1994]. Decay of mRNA ($d_m = 0.14$ hr^{-1}) is taken from Groenenboom et al. [2005] and decay of 'other' RNA is assumed to be twice as high ($d_r = 0.28$ hr^{-1}).

Finally, the stage of VLP production requires various proteins and processing steps. We assume it is the bottleneck of transposon dynamics, and hence we take $q = 1 \cdot 10^{-5}$ #mol^{-1} hr^{-1}.

Transcriptional gene silencing Parameters that are related to RNAi processes in the nucleus and that mirror RNAi in the cytoplasm (such as p_n and p_c) are taken to be equal to their cytoplasmic counterpart. See below.

To our knowledge estimates for the process of heterochromatin formation are not known (i.e. the time period that is needed for a gene or transposon to become packaged in heterochromatin). In order to arrive at a reasonable magnitude of the parameter values h_b, h_s and u, we perform the following estimation. We decompose heterochromatin formation into two steps: histone methylation and further packaging by proteins such as SWI6/HP1. First, enzyme kinetics of histone 3 lysine 9 (di)methylation, H3K9me, have been measured *in vitro* for the protein Dim5 of *Neurospora crassa*: a maximum turnover of $k_{cat} = 2.3$ min^{-1} = 138 hr^{-1} was observed [Gowher et al., 2005]. Second, if we take the length of a transposon 6000 base pairs, it contains ~ 30 nucleosomes that may be methylated. Each nucleosome has 2 histone H3 proteins, thus in the order of 60-120 (di)methylations prepare the TE for packaging into heterochromatin. This brings us to a maximum of 138/120 = 1.15 transposons per hour that become ready for transcriptional silencing. Next, SWI6/HP1 needs to bind the methylated nucleosomes. As a short-cut for determining how quickly a TE is packaged by SWI6/HP1, we take it to be in the same order of magnitude as histone methylation.

Due to other nuclear processes and crowding effects we expect that the maximum rate that we calculated here is usually not reached. Instead, we estimate the full process of heterochromatin formation should be in the range $[0.01, 0.10]$. This brings us to a basal heterochromatin formation rate of $h_b = 0.01$ hr^{-1} and activation – that is removal of SWI6/HP1 such that transcription may take place – by default twice as high ($u = 0.02$ hr^{-1}). Thus we assume a rather negative scenario from the view point of silencing: the genome prefers to be open and transcribed. Furthermore, siRNA induced heterochromatization is taken $h_s = 0.001$ #mol^{-1} hr^{-1}, that is in the same order of magnitude as RISC activity.

Post-transcriptional gene silencing The parameters have been taken from literature [Groenenboom et al., 2005, Kim and Yin, 2005]. Furthermore, dsRNA formation via mRNA and asRNA ($p_{cx} = 2 \cdot 10^{-4}$ #mol^{-1} hr^{-1}) we estimate as a rare event, in-between RISC cleavage ($b = 8 \cdot 10^{-3}$ #mol^{-1} hr^{-1}) and the assembly of a VLP ($q = 1 \cdot 10^{-5}$ #mol^{-1} hr^{-1}). dsRNA formation via hairpins is taken to be equal to RdRP activity ($p_{cxx} = 0.002$ hr^{-1}).

References

H Gowher, X Zhang, X Cheng, and A Jeltsch. Avidin plate assay system for enzymatic characterization of a histone lysine methyltransferase. *Anal Biochem*, 342(2):287–291, Jul 2005.

MAC Groenenboom, AFM Marée, and P Hogeweg. The RNA silencing pathway: the bits and pieces that matter. *PLoS Comput Biol*, 1(2):155–165, Jul 2005.

A. Jarmolowski, W. C. Boelens, E. Izaurralde, and I. W. Mattaj. Nuclear export of different classes of RNA is mediated by specific factors. *J Cell Biol*, 124(5):627–635, Mar 1994.

H Kim and J Yin. Robust growth of human immunodeficiency virus type 1 (HIV-1). *Biophys J*, 89(4): 2210–2221, Oct 2005.
